# Supramolecular aggregation of aquaporin‐4 is different in muscle and brain: correlation with tissue susceptibility in neuromyelitis optica

**DOI:** 10.1111/jcmm.13401

**Published:** 2017-10-20

**Authors:** Stefania Rosito, Grazia Paola Nicchia, Claudia Palazzo, Anna Lia, Cinzia Buccoliero, Francesco Pisani, Maria Svelto, Maria Trojano, Antonio Frigeri

**Affiliations:** ^1^ Department of Bioscience, Biotechnologies and Biopharmaceutic University of Bari “Aldo Moro” Bari Italy; ^2^ Dominick P. Purpura Department of Neuroscience Albert Einstein College of Medicine Bronx NY USA; ^3^ Department of Basic Medical Sciences, Neurosciences and Sense Organs University of Bari Aldo Moro Bari Italy

**Keywords:** neuromyelitis optica, aquaporin‐4, orthogonal arrays of particles, supramolecular structures, STED

## Abstract

Neuromyelitis optica (NMO) is an autoimmune demyelinating disease of the central nervous system (CNS) caused by autoantibodies (NMO‐IgG) against the water channel aquaporin‐4 (AQP4). Though AQP4 is also expressed outside the CNS, for example in skeletal muscle, patients with NMO generally do not show clinical/diagnostic evidence of skeletal muscle damage. Here, we have evaluated whether AQP4 supramolecular organization is at the basis of the different tissue susceptibility. Using immunofluorescence we found that while the sera of our cohort of patients with NMO gave typical perivascular staining in the CNS, they were largely negative in the skeletal muscle. This conclusion was obtained using human, rat and mouse skeletal muscle including the AQP4‐KO mouse. A biochemical analysis using a new size exclusion chromatography approach for AQP4 suprastructure fractionation revealed substantial differences in supramolecular AQP4 assemblies and isoform abundance between brain and skeletal muscle matching a lower binding affinity of NMO‐IgG to muscle compared to the brain. Super‐resolution microscopy analysis with g‐STED revealed different AQP4 organization in native tissues, while in the brain perivascular astrocyte endfoot membrane AQP4 was mainly organized in large interconnected and raft‐like clusters, in the sarcolemma of fast‐twitch fibres AQP4 aggregates often appeared as small, relatively isolated linear entities. In conclusion, our results provide evidence that AQP4 supramolecular structure is different in brain and skeletal muscle, which is likely to result in different tissues susceptibility to the NMO disease.

## Introduction

Aquaporin‐4 (AQP4), initially named MIWC (mercurial insensitive water channel), is an orthodox water channel abundantly expressed in the astrocyte cell plasma membrane, involved in the maintenance of cerebral water balance, blood–brain barrier development and integrity 1, 2. Recent studies also suggest a role for AQP4 in the glymphatic pathway by accelerating paravascular interstitial solute clearance 3. Nevertheless, the only illness in which AQP4 has been demonstrated to be directly involved is neuromyelitis optica (NMO), an autoimmune demyelinating disease of the central nervous system 4. A large majority of patients with NMO produce antibodies (NMO‐IgG) that specifically bind to AQP4 and trigger both complement‐ and antibody‐mediated cytotoxicity 5, 6. NMO has been defined as a glialpathology as it affects AQP4 function in astrocytes, mainly of the optic nerve and spinal cord, the two tissues where AQP4 is highly expressed in the CNS 7.

AQP4 is also expressed outside the CNS, for example in kidney, stomach and skeletal muscle 8, 9. However, patients with NMO generally do not show clinical/diagnostic evidence of tissue damage outside the CNS. Few hypotheses have been reported about the NMO tissue‐specific pathogenesis. The most interesting idea is that total AQP4 protein is higher in NMO susceptible tissues 7. A recent study also suggests that tissue‐specific complement regulators may protect peripheral organs but not the CNS 10 providing an alternative hypothesis on why NMO primarily damages the CNS. Other possibilities regard accessibility of AQP4 from non‐CNS tissues to NMO‐IgG, differences in the inflammatory microenvironment and different AQP4 interacting proteins.

A previous study on the molecular characterization of the major NMO‐IgG epitopes led us to suggest that tissue‐specific differences in AQP4 plasma membrane organization may also affect NMO‐IgG epitope formation and thus NMO pathogenesis 11.

Regarding the involvement of skeletal muscle in NMO, the little evidence available is related to hyperCKemia with a concomitant alteration of AQP4 in skeletal muscle reported in sporadic NMO patients 12, 13, 14. However, in a large retrospective study <1% of patients with NMO had prominent hyperCKemia 15. These data suggest that skeletal muscle function is mostly unaffected in patients with NMO. One possible explanation is that AQP4 in skeletal muscle may not have a physiological role 16 compared to that of the CNS and thus its alteration would not produce a clinical phenotype. However, the highly selective location of AQP4 at the sarcolemma membrane of fast‐twitch fibres 17 together with data on muscle activity in different conditions, such as unloading 18 and physical exercise 19, provides strong evidence of an important role for AQP4 in muscle physiology, associated with the metabolic glycolytic metabolism 20.

AQP4 is expressed as two major isoforms M1 and M23. Both AQP4 isoforms function as water channels, although their relative abundance is tissue specific 21, 22. The M1 and M23 isoforms reside within heterotetramers and their formation reflects the relative expression levels of the two principal isoforms. A peculiar characteristic of AQP4 is that these tetramers spontaneously aggregate, at the plasma membrane, to form very well‐ordered supramolecular structures called orthogonal arrays of particles (OAP) a two‐dimensional arrangement observable by freeze fracture electron microscopy (FFEM) 23. These OAPs have been identified in the plasma membrane of fast‐twitch skeletal muscle fibres 8, 24, 25, astrocyte endfeet 26, 27 and other AQP4‐expressing cells 28.

Several lines of evidence indicate that the heterotetrameric composition, and thus the ratio between M1 and M23, is the major factor in controlling the OAP size with the longer M1 isoform being the limiting factor. Indeed, the M1 isoform is incorporated in OAPs but only at the periphery, thus modulating OAP size 29. Recent studies indicate that a new AQP4 isoform, called AQP4ex, may modulate OAP size 30.

Although the functional role of OAPs is not understood, several pieces of evidence suggest that the low OAP plasma membrane diffusion rate could be an element to segregate the protein into a particular membrane domain as happens at the perivascular pole of astrocyte processes 31.

Importantly, it is now generally accepted that OAPs are the principal target of AQP4‐IgG 29, the pathogenic immunoglobulin produced by patients affected by NMO 32. As OAPs are expressed in astrocytes and fast‐twitch fibres, it is not obvious why skeletal muscle is much less affected in patients with NMO. Furthermore, previous studies have demonstrated the existence in the cerebellum of at least two independent AQP4 pools 33, 34 and that one, the perivascular, is the NMO related 29. Thus, the purpose of this study was (*i*) to evaluate to what extent NMO sera (AQP4‐IgG+) are able to recognize AQP4‐expressing skeletal muscle fibres *versus* AQP4 expressed in brain and (*ii*) to provide information on AQP4 isoform expression and OAP pool composition, tissue‐specific suprastructure organization and their effect on NMO epitope binding.

The results demonstrate that skeletal muscle AQP4 expression/composition is different to that of the CNS and this generates a distinctive supramolecular organization, which is likely to result in inefficient binding of NMO‐IgG to skeletal muscle AQP4.

## Materials and methods

### Human samples and NMO Sera

Human skeletal muscle samples (quadriceps) were obtained from patients who underwent surgery. Informed consent was obtained from the patients. Sera from fifty‐one patients with NMO were used for this study. All the sera were tested by an in‐house CBA method at the Laboratory of Neurochemistry of the Department of Basic Medical Sciences, Neuroscience and Sense Organs, University of Bari.

### Animals

Experiments were performed in accordance with the European directive on animal use for research. This project was approved by the Institutional Committee on Animal Research and Ethics of the University of Bari and by the Italian Health Department (Project no 100/2014‐B). Wistar rats were maintained under a 12‐hrs dark to light cycle, constant room temperature and humidity (22 ± 2°C, 75%), with food and water provided *ad libitum*, and they were supplied with environmental enrichment materials.

### Antibodies

Anti‐AQP4 was purchased from Santa Cruz Biotechnology (goat polyclonal, sc‐9888, Finnell ST 75220 Dallas, Texas) and diluted 1:100 for ELISA, 1:300 for immunofluorescence and 1:500 for immunoblotting and immunoprecipitation experiments 35, 36. The secondary antibodies used for Western blot were donkey anti‐goat (Santa Cruz Biotechnology, sc‐2020, Finnell ST 75220 Dallas, Texas) diluted 1:5000. The secondary antibody used for immunofluorescence was Alexa Fluor 488 anti‐goat (Molecular Probes, A‐11055 Eugene, Oregon, USA) diluted 1:1000.

### Plasma membrane vesicles from brain

Whole rat brains (five rats), including the cerebellum, were explanted and homogenized using a Potter apparatus in 5 volumes of ice‐cold homogenizing buffer (250 mM Sucrose, 10 mM Tris‐HCl, pH 7.5) with an added protease inhibitor cocktail (Roche Diagnostics, Indianapolis, Indiana, USA www.roche.com) for protein stability. The homogenate was centrifuged at 800 × *g* for 10 min. at 4°C, and the supernatant was first centrifuged at 8,000 × *g* for 10 min. and then centrifuged at 17,000 × *g* for 45 min. at 4°C to obtain a low speed pellet enriched in AQP4‐containing plasma membrane vesicles 34.

### Preparation of rat sarcolemma vesicles

Sarcolemma vesicles were prepared using a procedure that has previously been described elsewhere 20. Briefly, approximately 450 g of rat leg muscles (from ten 6‐month‐old rats) was excised and homogenized in 7.5 vol of a buffer containing 20 mM sodium pyrophosphate, 20 mM sodium phosphate monohydrate, 1 mM MgCl_2_, 0.303 M sucrose and 0.5 mM EDTA, pH 7.0, in the presence of a cocktail of protease inhibitors. The homogenate was centrifuged at 14,000 × *g*, and the supernatant centrifuged for 30 min. at 30,000 × *g*, and the resulting supernatant (S30) was used to isolate light microsomes (LMs). Solid KCl to a final concentration of 0.6 M was added to the S30, and the suspension was then centrifuged for 35 min. at 142,000 × *g*. The pellets were re‐suspended in 400 ml of a buffer containing 0.303 M sucrose, 50 mM Tris‐HCl, pH 7.4, treated again with KCl and centrifuged as before. The pellets were finally re‐suspended with the same buffer, and the protein concentration was determined.

### Native size exclusion chromatography (nSEC)

Five different extraction conditions were first tested. Proteins from vesicles were extracted on ice for 1 hr, vortexed every 5 min., in 7 volumes of Extraction Buffer (500 mM aminocaproic acid, 50 mM imidazole, 2 mM Ethylenediaminetetracetic acid (EDTA), 3% n‐Dodecyl β‐D‐maltoside (DDM) and protease inhibitor cocktail) added with 12 mM or 150 mM NaCl, and with 0%, 2% or 10% glycerol, depending on the assay. The protein lysate was centrifuged at 22,000 × *g* for 30 min. at 4°C, and the supernatant was used for immunoprecipitation and nSEC experiments.

nSEC was performed as previously described 35. Briefly, rat brain and muscle plasma membrane vesicles were extracted in nSEC‐Extraction buffer (Extraction buffer with 150 mM NaCl added), and the protein content was measured by BCA protein assay (Thermo). Lysate was then injected into AKTA‐FPLC using one of two types of stationary phase, Sephacryl S‐500 high prep 16/60 or 26/60 (GE Healthcare Life Sciences, Little Chalfont, UK). All chromatographic phases were performed at room temperature, max 0.15 MPa of column pressure and 1 ml/min. of flux rate. Columns were first equilibrated with 2 column volumes of nSEC‐buffer‐0.15% DDM (500 mM aminocaproic acid, 50 mM imidazole, 2 mM EDTA, 0.15%DDM, 150 mM NaCl) and then injected with 1 or 5 ml of protein lysate (10 mg/ml) in 16/60 and 26/60 columns, respectively. The absorbance at 280 nm was continuously monitored and fractions of 3 ml for the 16/60 column or 8 ml for the 26/60 column were collected. Total protein content of nSEC fractions was quantified using a Micro‐BCA Protein Assay Kit (Thermo).

### Dot blot

After the nSEC, the AQP4 elution profile was evaluated by dot blot. Two microlitres of each fraction was spotted onto a nitrocellulose membrane blocked with 5% milk in 1% Triton X‐100in PBS and processed as reported for regular AQP4 immunoblotting.

### SDS‐PAGE and western blot analysis

Membrane proteins were dissolved in Laemmli Sample Buffer (Bio‐Rad, Hercules, California, USA) and 50 mM dithiothreitol, heated to 37°C for 10 min., resolved in a 13% polyacrylamide gel and transferred onto PVDF membranes for immunoblot analysis.

After transfer, the membranes containing the blotted proteins were blocked with milk 5% in PSB‐1%Triton and incubated with primary antibodies diluted as described in the Antibodies section. After washing, the membranes were incubated with peroxidase‐conjugated secondary antibodies and washed again. Reactive proteins were revealed with an enhanced chemiluminescent detection system (ECL‐Plus; Pierce Euroclone, Milan, Italy) and visualized on a VersaDoc imaging system (Bio‐Rad).

### Blue native (BN) and two‐dimensional BN/SDS‐PAGE

Twenty‐five microlitres of eluted fractions (about 20 μg) were mixed with 5% CBB G‐250 (Coomassie blue G‐250) and loaded in polyacrylamide native gradient gels (3–9%) as previously described 34. After electrophoresis, proteins were blotted onto polyvinylidenedifluoride (PVDF) membranes (Millipore, Milan, Italy) for immunoblot analysis. For 2DE, the lanes from the first dimension were cut into individual strips, equilibrated in denaturation buffer (1% SDS and 1% β‐mercaptoethanol) and placed into a 2D SDS–PAGE of the same thickness. The second dimension was performed according to standard protocols. At the end of the run, the gel was blotted onto a PVDF membrane for Western blot analysis.

### Densitometry analysis

Images were analysed using ImageJ software (National Institutes of Health, Bethesda, MD). For relative quantification, the optical density value was determined for equal‐sized boxes that were drawn around antibody‐stained bands, and the background values were obtained below each band of interest to account for non‐specific antibody staining in the lane.

### Sandwich OAP‐ELISA

The ELISA was performed as previously described by Pisani *et al*., 35. Briefly, Maxisorp NUNC Plates (Thermo) were coated with 0.2 μg of commercial goat anti‐AQP4 antibody (Santa Cruz Biotechnology, sc‐9888) overnight at 4°C or for 2 hrs at 37°C. After coating, wells were washed and coated with approximately 35 ng of AQP4. Negative control wells were only coated with the buffer. After incubation for 1 hr, sera diluted from 1:1000 to 1:8000 and goat anti‐AQP4 were incubated in the wells for 1 hr under shaking. Wells were then washed, incubated with anti‐human biotinylated secondary antibody (Millipore AP112B), washed again and incubated with streptavidin‐HRP (Millipore SA202, 1:1000 in A). After 1 hr, 100 μl of TMB solution was added (Millipore) for 20 min., and the reaction was stopped by adding 100 μl of 0.3M sulphuric acid solution. Finally, absorbance was read at 450 nm. Normalized absorbance was calculated as follows: absorbance of AQP4‐coated well minus absorbance of negative control well. Absorbance was read using a Flex Station 3 (Molecular Devices, Sunnyvale, California, USA).

### Immunofluorescence on tissues sections

Eight micrometres transverse sections of frozen tibialis anterior and brain tissues were prepared using a cryostat (CM 1900; Leica) air‐dried for 15 min. and stored on positively charged glass slides (Thermo Scientific, Waltham, Massachusetts, USA) at −80°C. When needed, sections were thawed, fixed in 4% formaldehyde, blocked using 0.1% gelatin in PBS for 30 min. at room temperature (RT) and then incubated at RT for 1 hr with primary antibodies, washed with PBS‐gelatin and incubated with Alexa‐conjugated secondary antibodies. Sections were washed and finally mounted with Mowiol or PBS‐glycerol, pH 8.0, containing 1% n‐propyl gallate. In the case of NMO‐IgG staining, tissue sections were only fixed at the end of the staining procedure to preserve conformational epitope integrity as previously reported 36.

### STED imaging

Tissue sections were analysed by g‐STED as previously described 30. The system that was used consisted of a Leica TCS SP8 3X fully automated epifluorescence confocal microscope, with AOTF and AOBS, white light laser (WLL2), gated HyD detection, gated STED 3D and two depletion lasers (594 nm; 660 nm). Excitation of Alexa Fluor 488 dye was performed with the laser at 488 nm. STED was performed with the depletion laser emitting at 594 nm. The excitation beams and the doughnut‐shaped STED beam were co‐aligned and coupled into a 100×/1.40 Oil STED White objective. Image analysis was performed with LAS AF LITE software, drawing ROIs around stained spots and clusters, which were quantified by their full width at half maximum (FWHM) intensity. Huygens professional software (Scientific Volume Imaging, Laapersveld, VB Hilversum, Nederland) was used for STED image deconvolution and to determine the aggregate size of AQP4 30.

### Statistical analysis

All data are reported as mean ± S.E.M. of three or more experiments. We used the Student's *t*‐test for unpaired data and analysis of variance for multiple statistical comparisons between groups. Differences were considered significant for *P* < 0.05.

## Results

### Immunofluorescence comparative analysis of NMO‐IgG binding to brain and skeletal muscle

**Figure 1:**
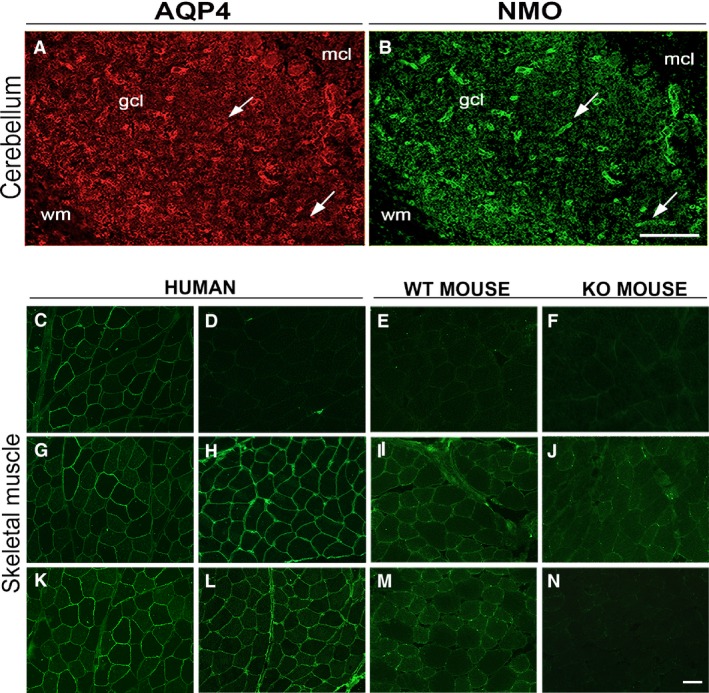
NMO‐IgG immunofluorescence analysis of rat cerebellum and skeletal muscle. Top, staining of the cerebellum with AQP4 (**A**) and NMO‐IgG (**B**). gcl, granular cell layer; mcl, molecular cell layer. Arrows indicate staining of pericapillary astrocyte endfeet. Note the selective labelling of the NMO‐IgG to the astrocyte endfeet processes. Bottom, staining of AQP4 and NMO‐IgG of the skeletal muscles. (**C, G and K)** show human muscle sections stained with AQP4 commercial antibodies. (**D–F)** staining with an NMO serum that gave no signal. (**H–J**) staining with an NMO serum that produced a non‐specific signal (positive in both WT and AQP4‐KO mice). (**L–N**) staining with an NMO serum that presented a specific stain. Note, in this latter case, the absence of staining in skeletal muscle from AQP4‐KO mouse (**N**). Scale bar, 50 μm.

Immunofluorescence experiments were performed to evaluate tissue‐specific differences in the binding capacity of NMO sera to AQP4. To this purpose, sera from AQP4‐IgG‐positive patients (*n* = 51) were used and tested in parallel on rat brain and skeletal muscle using unfixed frozen sections. As previously reported 29, NMO sera preferentially stain the perivascular pool (dystrophin‐dependent) of AQP4 therefore implying the presence of AQP4 suprastructures with different binding affinity for NMO‐IgG (Fig. 1A and B). Interestingly, while all NMO sera were able to stain mouse brain astrocyte processes close to capillaries, the skeletal muscle fibres were largely not stained by the NMO sera (Fig. 1C–N). Skeletal muscle from human biopsies and from WT and AQP4‐KO mouse was used to determine both the specificity of the staining, and whether species‐specific differences could occur. Immunofluorescence results revealed that the large majority (30) of the NMO sera were either negatives (Fig. 1D–F) or produced (19 sera) a non‐ specific stain (positive in WT and AQP4‐KO mice) of the skeletal muscle fibres (Fig. 1H–J). Only two NMO sera were able to stain exclusively the fibre sarcolemma, although faintly (Fig. 1L–N). Interestingly, the analysis of the liquor of these two patients revealed strong positivity on transfected cells and staining of CNS/skeletal muscle indicative of the presence of NMO‐IgG antibodies. Purified AQP4‐IgG antibodies, using transfected cells, from these two cases showed staining of skeletal muscle fibres, again confirming the specificity of the staining (not shown).

### Biochemical analysis of AQP4 organization in brain and skeletal muscle

**Figure 2:**
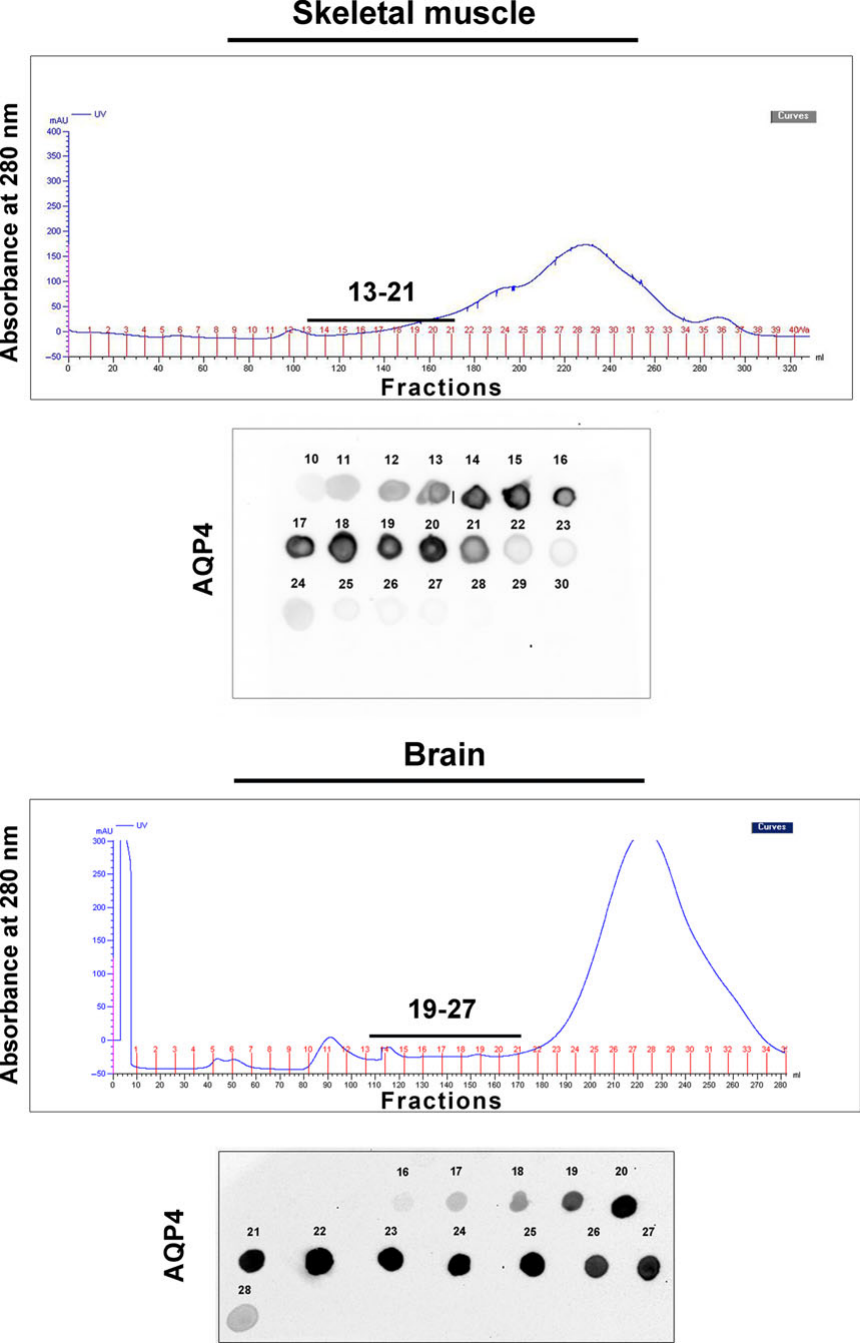
Muscle and brain OAPs separation by nSEC. For each tissue indicated, the typical chromatograms (top part) obtained from skeletal muscle and brain vesicles lysates and the dot blot analysis (bottom part) for the evaluation of AQP4 levels in the eluted fractions. Note the different elution profile of AQP4.

To elucidate the origin of the inadequate NMO‐IgG binding to skeletal muscle, the supramolecular organization of AQP4 was initially evaluated by performing high‐resolution size exclusion chromatography. Rat brain (including the cerebellum) and skeletal muscle membrane vesicles lysates were subjected to nSEC separation, and the AQP4 level in each eluted fraction was estimated (Fig. 2) by dot blot analysis. Differences in the AQP4 elution profile were observed: skeletal muscle AQP4 was eluted in an earlier fraction interval (13–21) compared to the brain (19–27) extracts suggesting a different AQP4‐OAPs pattern profile in the two tissues.

**Figure 3:**
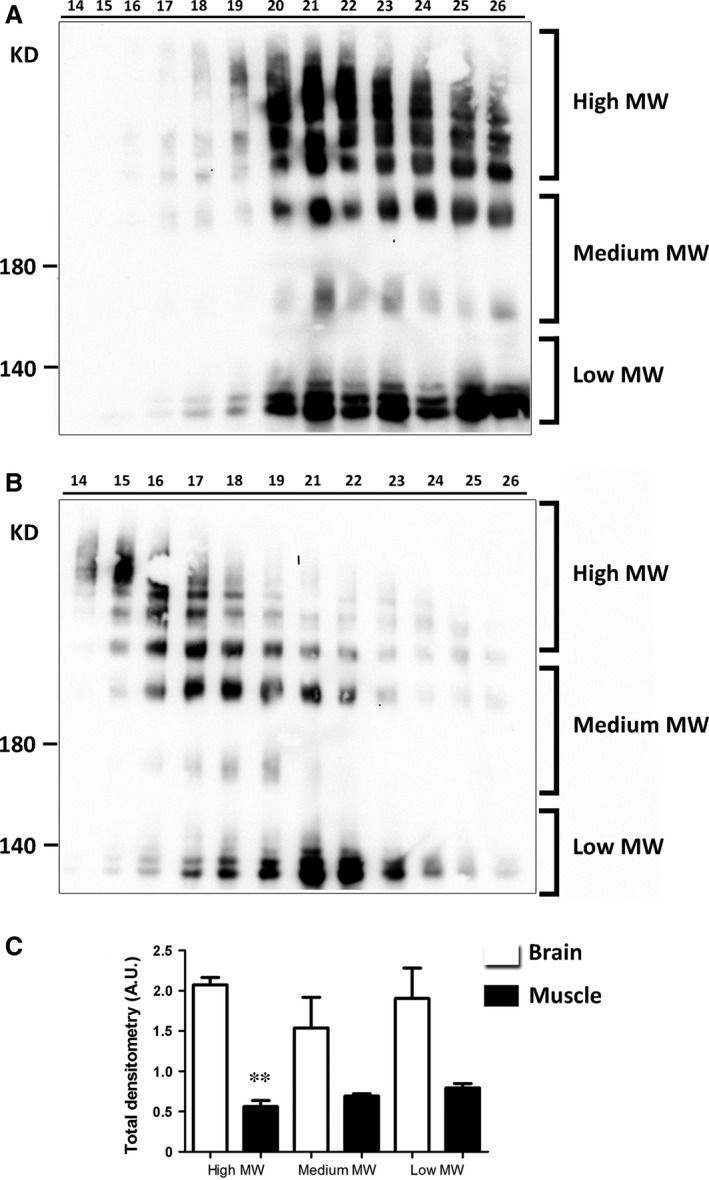
Analysis of AQP4 pools by BN‐PAGE. (**A, B**) AQP4 immunoblot of nSEC fractions from both whole brain (**A**) and skeletal muscle (**B**), after a 3–9% gradient BN gel. Note the presence of several AQP4 pools and the different elution profile of AQP4 in the two tissues. Depending on their position on the blot, AQP4 pools were classified as high, medium or low molecular weight pools. (**C**) Densitometry analysis of the BN‐PAGE immunoblot results to evaluate the relative abundance of the three AQP4 pools in the two tissues (*P < 0.01, *n* = 3. anova test). Similar protein loading in each lane was evaluated by Coomassie blue staining of the PVDF membrane.

A BN‐PAGE experiment was then performed to evaluate the profile of AQP4 pools in nSEC fractions from both tissues (Fig. 3). Several pools of AQP4 were visible as individual spots in immunoblot analysis and, based on their approximate size, AQP4 pools were divided into three groups: high, medium and low molecular weight (MW) pools. The electrophoretic profile showed a larger amount of high MW pools in brain fractions compared to the skeletal muscle fractions. Densitometry analysis suggested a significant reduction in the absolute AQP4 high MW pool level in fractions derived from the muscle.

**Figure 4:**
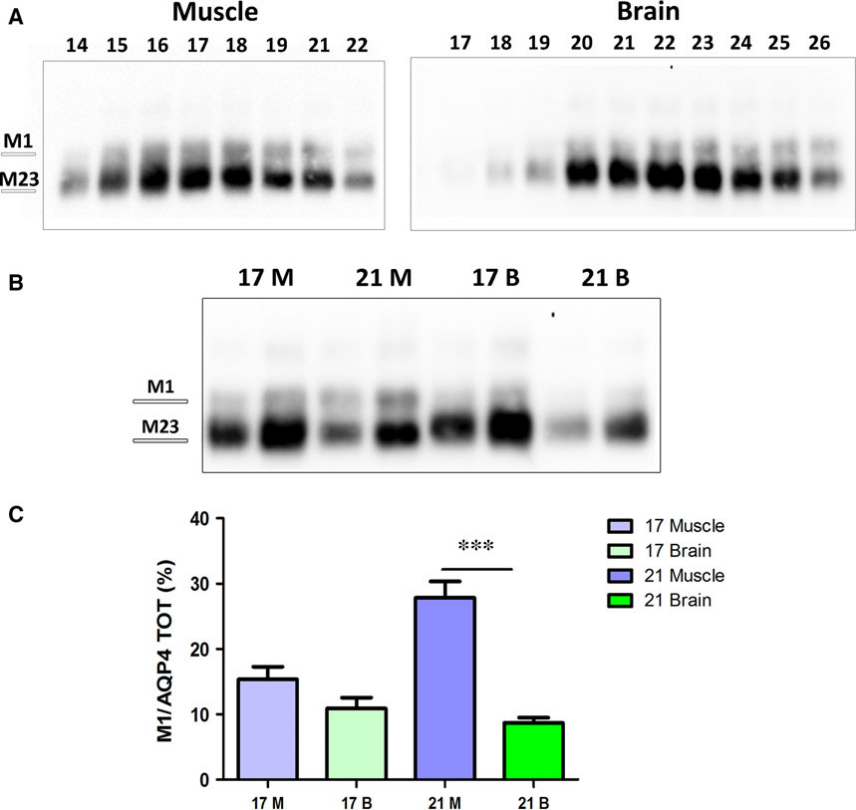
AQP4‐M1 levels in nSEC fractionation. (**A**) Immunoblot after SDS‐PAGE of the eluted fractions obtained from skeletal muscle and brain. (**B**) Immunoblot of fraction 17 and 21 from muscle (M) and brain (B). Two protein concentrations were used for each fraction, and the amount loaded in each fraction was calibrated to better evaluate the level of the two isoforms in each fraction. (**C**) Densitometry analysis of M1 levels in the selected fractions. (***P < 0.0001, *n* = 4).

Orthogonal arrays of particles (OAP) size depends on the expression ratio of the M1 and M23 isoforms, both of which are required for AQP4 supramolecular organization in native tissues 22, 34, with M1 having a role in reducing OAP size. We thus analysed the proportion of M1 in each nSEC fraction of both tissues (Fig. 4). Interestingly, the M1 isoform was progressively more abundant in later fractions (more retained), consistent with BN‐PAGE data that showed an enrichment of increasingly smaller AQP4 aggregates in those fractions. Moreover, the M1 isoform was more abundant in all the skeletal muscle‐derived fractions compared with the brain fractions indicating that M1 could represent an important determinant in the tissue‐specific AQP4 supramolecular organization. To better evaluate the contribution of the two isoforms in the pool formation and relate it to possible functional/pathological consequences, we evaluated two fractions (17 and 21) for each tissue with a different content of AQP4 pools: one characterized by mostly high MW pools (fraction 17) and one containing more heterogeneous AQP4 pools (fraction 21). Immunoblot analysis after SDS‐PAGE revealed that the M1 isoform content was only significantly different in fraction 21, being about three times higher in skeletal muscle compared with brain (Fig. 4B and C).

**Figure 5:**
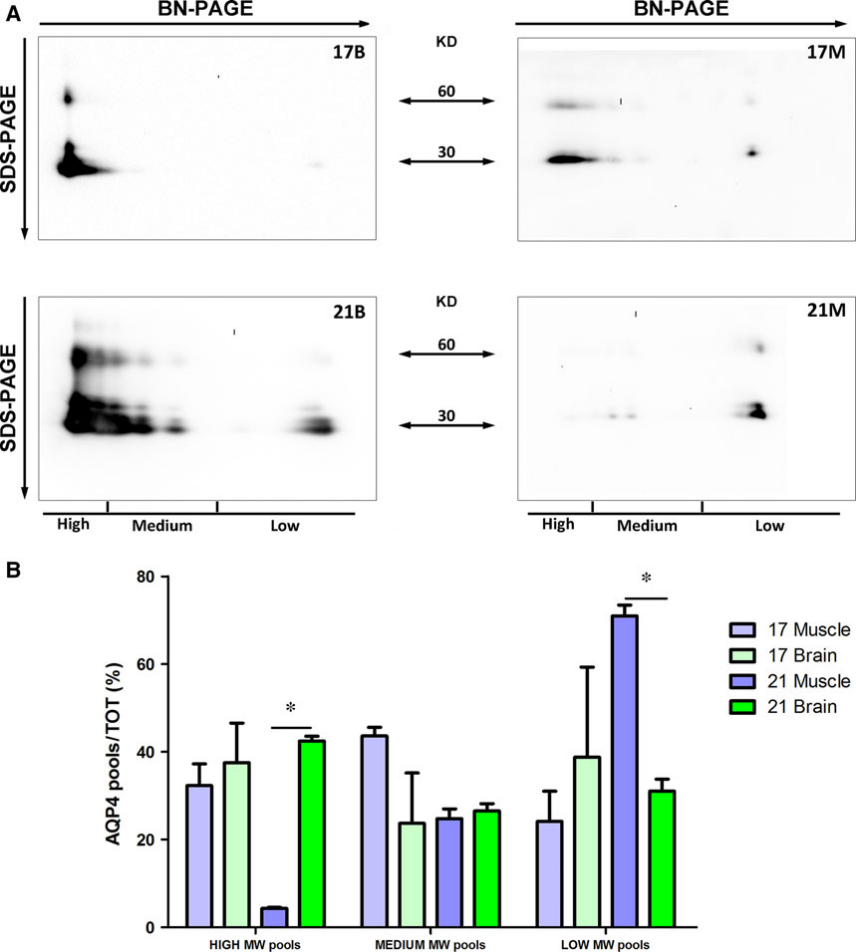
Analysis by 2DE of AQP4 pool composition in selected brain and skeletal muscle fractions. (**A**) Immunoblot after BN/SDS‐PAGE of fractions 17 and 21 from brain (B) and muscle (M). (**B**) Analysis of high, medium and low molecular weight (MW) AQP4 pools in the selected fractions. AQP4 pool percentages were determined by densitometry analysis of each pool group normalized on total AQP4 amount. Note the statistically significant differences in fractions 21 and 17 (*P < 0.05, *n* = 3–4).

A more detailed analysis of the two fractions was performed by 2D electrophoretic (2DE) analysis (Fig. 5). As high MW pools of AQP4 transferred poorly on the immunoblot membrane after BN‐PAGE, immunoblot of 2DE provided more accurate information on the AQP4 pool composition. For the first dimension, AQP4 complexes were separated by BN‐PAGE (3–9% gradient) while the second dimension was performed in SDS‐PAGE (12%). 2DE analysis revealed a different profile of the muscle and brain fractions. In the two muscle fractions, low MW AQP4 pools appeared more abundant than in the two brain fractions, in which, instead, it revealed high MW AQP4 pools. Densitometry analysis of the immunoblot results revealed statistically significant differences in fraction 21 (Fig. 5B). In muscle‐derived fraction 21, we observed a virtual absence of high MW AQP4 pools and an abundance of low MW pools.

These data confirm that the content of AQP4 isoforms controls the size and distribution of AQP4 pools and results in the different elution profile of the two tissues.

**Figure 6:**
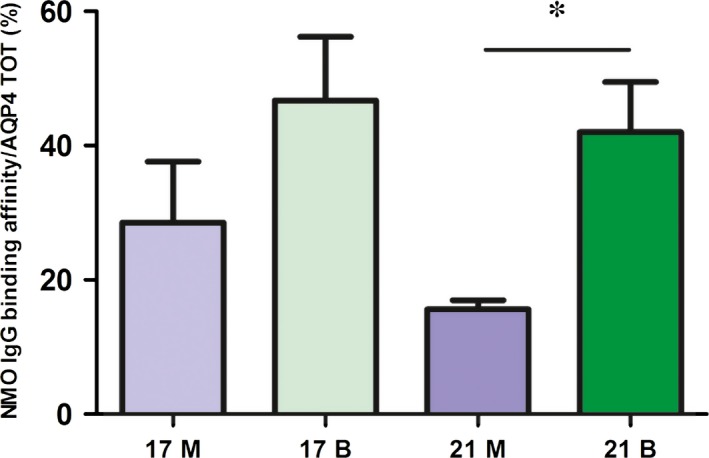
Analysis of NMO‐IgG binding affinity to nSEC fractions. Eluted fractions were tested for the commercial anti‐AQP4 antibody and NMO serum in ELISA experiments (see Materials and methods). NMO‐IgG affinity to AQP4 was calculated as the protein amount recognized by NMO‐IgG compared to total AQP4 for each selected fraction. (*P value < 0.05; *n* = 3).

We then tested whether NMO‐IgG affinity is correlated to AQP4 pool composition and in particular to a lower abundance of those of higher MW in the skeletal muscle. Then, the four fractions were tested for NMO‐IgG binding by ELISA. The results (Fig. 6) show lower NMO‐IgG affinity for fraction 21 derived from skeletal muscle compared to the same fraction of brain, suggesting that the reduced NMO‐IgG affinity to the muscular AQP4 could be related to a lesser amount of high size AQP4 pools. Furthermore, NMO‐IgG binding affinity to AQP4 decreased with the progression of the elution (not shown).

### Super‐resolution microscopy analysis of AQP4 organization in native tissues

The biochemical analysis of AQP4‐expressing tissues allowed us to identify some potentially important elements in NMO tissue‐specific pathogenesis. However, collecting the best information regarding AQP4 membrane organization required it to be analysed in its natural context. To this purpose, we evaluated AQP4 supramolecular structures in cerebral perivascular astrocyte endfoot and at the sarcolemma of rat skeletal muscle fast‐twitch fibres using g‐STED microscopy, which allowed us to determine the size and distribution of individual AQP4 clusters at the nanoscale level.

**Figure 7:**
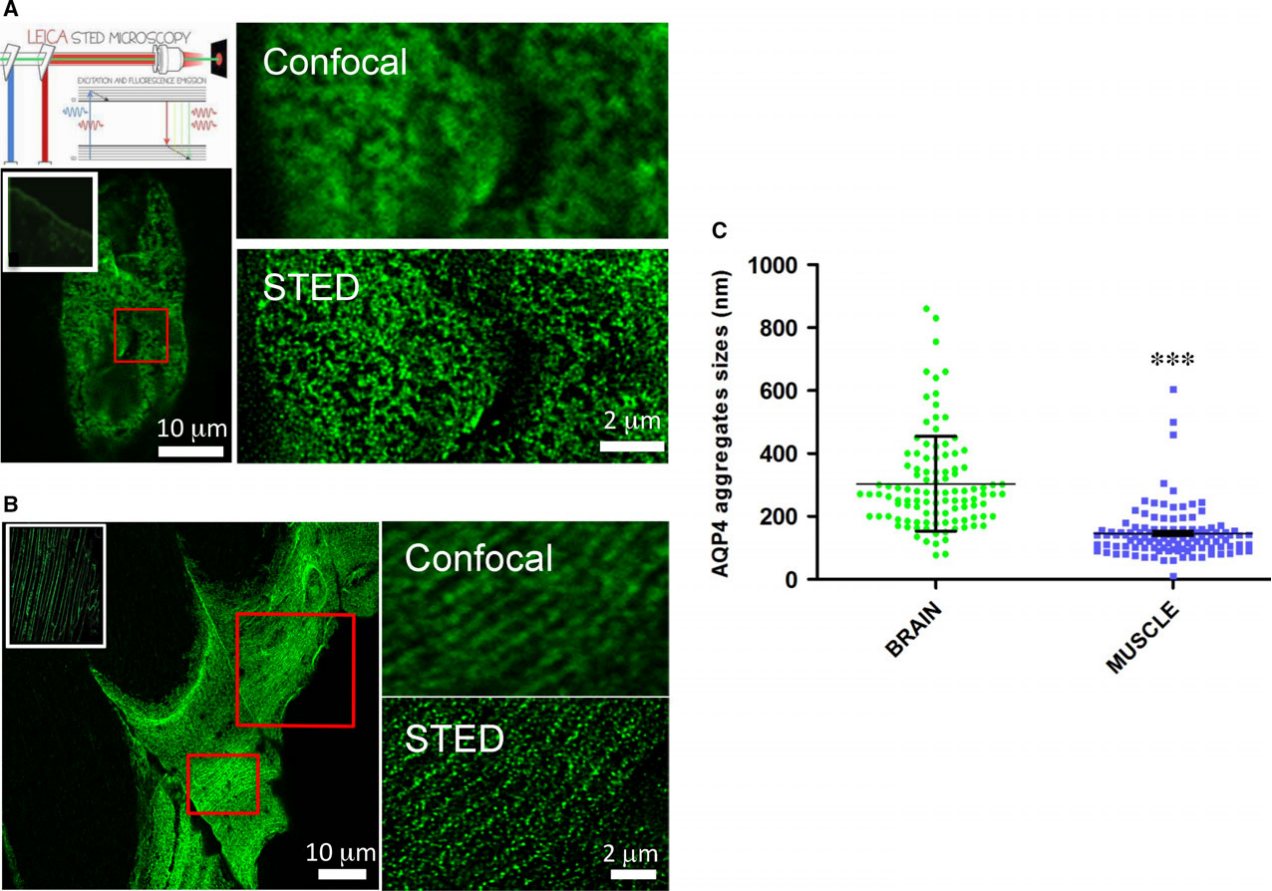
AQP4 supramolecular organization in rat brain cortex and skeletal muscle. (**A**) Top left, schematic diagram of the principle of STED microscopy (Leica). Two lasers are used for STED: excitation is performed with the laser at 488 nm while STED is performed with the depletion laser emitting at 594 nm. The excitation beams and the doughnut‐shaped STED beam are co‐aligned and coupled into a 100× objective. Bottom left, brain blood vessel fragment stained with commercial anti AQP4 antibody and imaged using confocal microscopy. The typical staining of AQP4 astrocyte processes surrounding brain cortex capillaries is shown in the white box. Top right, magnification of the red‐boxed region by confocal (top) and g‐STED imaging. (**B**) AQP4 staining of a longitudinal section of the tibialis anterior muscle of rat. Left, tangential section through the sarcolemma of a fast‐twitch muscle fibre imaged with conventional confocal microscopy. The white‐boxed region shows a low magnification of AQP4‐stained fibres. Right, magnification of the red‐boxed region by confocal (top) and g‐STED imaging (bottom). (**C**) Analysis of AQP4 clusters in brain cortex and skeletal muscle. Left, scatter plot of AQP4 cluster sizes measured from the intensity profiles by the full width at half maximal intensity (FWHM). At least 50 random clusters were measured derived from 4–5 different portions. Performed statistical analysis: Student's *t*‐tests (*P* value < 0.0001).

Confocal images of tangential glial endfeet processes close to cerebral blood vessels (Fig. 7) show the AQP4 staining consisted of large closely‐spaced clusters which form structures similar to rafts. The use of g‐STED substantially increases image resolution in which the raft‐like clusters are much better defined and constituted of aggregations of well‐defined simple units.

Super‐resolution microscopy was also applied to fragments of sarcolemma from the fast‐twitch tibialis anterior muscle. In tangential sections, many of the large AQP4 clusters, observed with conventional confocal microscopy, were actually multiple smaller AQP4 clusters in close proximity (Fig. 7). Furthermore, in many areas, AQP4 appeared to be organized in linear structures, similar to lamellae arranged along a plane parallel to the long axis of the myofibre. In g‐STED, lamellae appear to consist of a greater number of aligned smaller clusters. This structural organization resembles the costamere organization of other sarcolemma proteins including the dystrophin‐glycoprotein complex (DGC).

Quantitative analysis (Fig. 7) showed that AQP4 clusters were about 2.5 times smaller in muscle fibres compared to those found in perivascular astrocyte endfeet. Furthermore, size frequency distribution revealed that brain cluster size falls into a wider range, while AQP4 clusters in muscle fibres are more homogenous in size and not very large. These results demonstrate different *in situ* AQP4 supramolecular organization.

## Discussion

It is now generally accepted that AQP4 supramolecular organization in OAPs is the central issue in NMO pathogenesis. One direct potential implication of this concept is its relationship with the different AQP4 tissue susceptibility in patients with NMO. Therefore, we evaluated whether tissue‐specific structural differences of OAP may explain this issue. Importantly, we found that the sera of our cohort of patients with NMO were mainly negatives in skeletal muscle, supporting the concept that the absence of damage to skeletal muscle in patients with NMO is merely due to the lack of binding of NMO‐IgG to muscle AQP4.

To assess AQP4 assembly more accurately in different tissues, we used a biochemical approach for OAP fractionation in their native conformation and accomplished a comparative analysis of AQP4 supramolecular structures in brain and skeletal muscle. nSEC fractionation and analysis by 2DE revealed substantial tissue differences in supramolecular AQP4 assemblies and isoform abundance. Furthermore, ELISA experiments indicate a lower binding affinity of NMO‐IgG to muscle compared to the brain. These differences are related to dissimilarities in large size AQP4 pools and in M1 isoform content between the two tissues. Previously, the M1 impact on NMO‐IgG affinity to its target was assessed in transfected cells 29, 37, 38. The biochemical analysis in native tissues supports a role for M1 in determining OAP size 23, 34, and indirectly points to the potential role of M1, together with M23, in the susceptibility of different tissues to the disease. However, we cannot exclude that other AQP4 isoforms, or interacting proteins, may also play a role in NMO‐IgG binding. Concerning this issue, a recent study reports the identification of an extended form of AQP4 (AQP4ex) generated by a translational readthrough mechanism 30. Future studies will address the role of this isoform in skeletal muscle AQP4 clustering and NMO‐IgG binding. Finally, we cannot exclude that the biochemical analysis of AQP4 supramolecular organization could be limited, because it requires the extraction of the protein from its natural context. However, what is significant is that AQP4 suprastructure is different in the analysed tissues. Although TIRFM was previously widely used for AQP4 microscopy analysis 29, resolution of OAPs was hampered by the diffraction limit of the technique (300 nm). Super‐resolution PALM‐microscopy has recently been used to evaluate AQP4 organization in cell models or primary cells, elucidating some structural features and molecular determinants of OAP assembly, but leaving largely unexplored AQP4 assembly in native tissues 39.

A few, mainly qualitative, data have been reported about AQP4 organization in skeletal muscle. An estimated size of 200–400 nm AQP4 clusters and occasional dot aggregates have been reported using confocal imaging analysis 40 but these data were diffraction limited. Here, we have used, for the first time to our knowledge, g‐STED microscopy to evaluate AQP4 organization at high resolution and in native tissues. Our data indicate that AQP4 clusters are markedly different between skeletal muscle fibres and perivascular astrocytes: being numerous, small and isolated in skeletal muscle fibres. g‐STED imaging improved optical resolution and allowed structures with an average size of approximately 80 nm (FWHM) in muscle fibres and approximately 190 nm (FWHM) in brain perivascular astrocytes to be visualized. Additionally, using g‐STED imaging, it has been possible to assess AQP4 cluster organization. In fast‐twitch fibres, AQP4 aggregates often appear as relatively isolated and linear entities, while in the brain perivascular region AQP4 is mainly organized in large interconnected and raft‐like clusters. This distinct membrane organization parallels the differences observed with biochemical analysis and are consistent with the different roles of M23 and M1 in aggregate formation. Interestingly, this structural organization observed in skeletal muscle resembles the costamere organization of other sarcolemma proteins including the dystrophin‐glycoprotein complex (DGC) and α‐syntrophin 41, 42. As the expression of α‐syntrophin is a critical factor in determining AQP4 localization at the sarcolemma of fast‐twitch fibres 43, 44, it could be expected that AQP4 at the costameres is the fraction of AQP4 associated with α‐syntrophin. This suggests that AQP4 spatial organization together with that of DGC belongs to a specific membrane domain to support myofibre structure and function. More importantly, g‐STED provides additional evidence to support the conclusion that AQP4 supramolecular organization is the main determinant for NMO‐IgG binding and further supports the concept that AQP4 monomer and tetramers do not contain the NMO‐IgG epitope. In addition, differences in AQP4 cluster size between different anatomic districts provide the first important indication of tissue‐specific AQP4 supramolecular organization as a crucial element capable of explaining the different behaviour of NMO‐IgG to its target in different body compartments. According to the literature, these structural differences should be due to the ratio of M1/M23 in tissues. However, the existence of a complex network of AQP4 and OAP regulating mechanisms suggests the need to investigate new potential players.

Our data also suggest that although other local factors (*i.e*. complement activators/inhibitors) may contribute to the tissue‐specific damage of NMO‐IgG, differences in AQP4 suprastructure are *per se* sufficient to explain the absence of skeletal muscle damage and provide a rationale as to why the effect is mainly in the CNS. It is also interesting to add that these structural differences in AQP4 plasma membrane assembly may have distinct functional relevance such that they may occur in the cerebellum granular cell layer where AQP4 is differently organized in the pericapillary (dystrophin‐dependent) site and in the intense astrocyte network (dystrophin‐independent). Interestingly, our study reveals the existence, in skeletal muscle, of a third type of AQP4 suprastructure, characterized by dystrophin‐complex dependence but which is not the target of NMO‐IgG.

In conclusion, our results provide additional evidence that the different binding affinity of NMO‐IgG to muscular and cerebral AQP4 is due to the diverse tissue‐specific AQP4 supramolecular organization, which may result in the different susceptibility of tissues to the disease.

## Conflict of interest statement

The authors confirm that there are no conflicts of interest.
